# Enzymatic synthesis of glycosides: from natural *O*- and *N*-glycosides to rare *C*- and *S*-glycosides

**DOI:** 10.3762/bjoc.13.180

**Published:** 2017-09-05

**Authors:** Jihen Ati, Pierre Lafite, Richard Daniellou

**Affiliations:** 1 ICOA UMR CNRS 7311, University of Orléans, rue de Chartres, BP 6759, 45067 Orléans cedex 2, France

**Keywords:** enzyme, glycochemistry, glycoside hydrolase, glycosyltransferase, mechanism

## Abstract

Carbohydrate related enzymes, like glycosyltransferases and glycoside hydrolases, are nowadays more easily accessible and are thought to represent powerful and greener alternatives to conventional chemical glycosylation procedures. The knowledge of their corresponding mechanisms has already allowed the development of efficient biocatalysed syntheses of complex *O*-glycosides. These enzymes can also now be applied to the formation of rare or unnatural glycosidic linkages.

## Introduction

The role of glycoconjugates is of prime importance, as they are nowadays well known to mediate many biological processes [1]. As a consequence, in a recently published roadmap for glycosciences in Europe, carbohydrates are expected, both by academics and industrials, to become key players in a near future in tremendous fields such as pharmaceuticals and personalized biomedicine, food, materials and renewable resources, and bioenergy for examples [2]. To achieve this goal, the glycoscientists will have to collaborate strongly to obtain pure and well-defined glycoconjugates. Indeed, even if during the last century, the chemists have engaged great efforts to successfully develop efficient means of synthesis of carbohydrate derivatives, through the use of specific protecting and/or activating groups and the fine control of the resulting anomeric linkage, thus leading now to i) a huge repertoire of stereoselective methods for glycosylation reactions [3] and ii) the premise of few automated oligosaccharide synthesis [4], such glycosylation process still remains highly target-dependent and therefore a challenge in too many cases. Even so, glycochemists were very recently able to chemically synthesize the largest polysaccharide to date: a mycobacterial arabinogalactan of 92 monosaccharide units [5]. However, recent advances especially in the area of molecular biology have allowed the emergence of biocatalytic procedures. Enzymes have proven to be efficient synthetic tools for the eco-compatible synthesis of many classes of compounds. Non-organic solvents, mild experimental conditions, and high regio- or stereospecificity of the biocatalysed reaction have increased the added value of the use of enzymes in transformation processes, from the laboratory bench to the industrial scale [6]. Moreover, genetic modifications of recombinant enzymes are now powerful tools to easily alter the versatility, as well as the properties of the engineered protein. Rational mutagenesis, directed evolution, or even de novo design have dramatically broaden the applicability of enzymes in biocatalysis [7]. In the glycochemistry field, a vast array of carbohydrate-metabolizing enzymes [8] has been used to synthesize glycosides, even using multiple enzymes systems. Glycoside hydrolases (GHs) or glycosyltransferases (GTs) have been focused on in the search for glycosylation tools, and have been extensively studied for genetic engineering [9–10]. The corresponding compounds have proven useful in many applications ranging from glycosylation of natural products to pharmaceutics [11]. Classically, glycosides are linked to the aglycone moiety through an oxygen or a nitrogen atom, although many other kinds of linkages (even if rare) can be found in nature like in glycosylated proteins for example [12]. Herein, we wish to report a short but comprehensive review of the current enzymatic methods described for the synthesis of unusual *C*- and *S*-glycosidic linkages, their mechanisms and the corresponding perspectives.

## Review

### Glycosyltransferases

**Figure 1:**
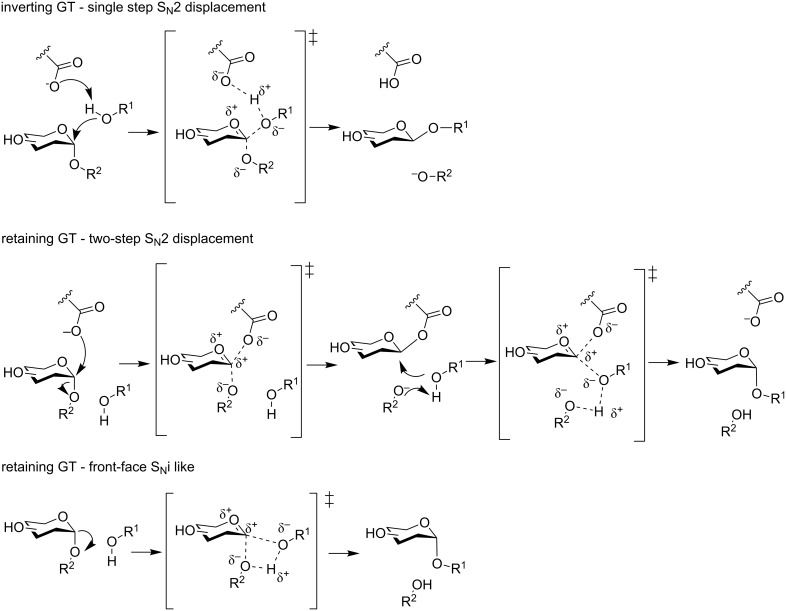
Mechanisms of *O*-GTs-catalysed glycosylation.

Glycosyltransferases (GTs, E.C. 2.4.1.x) catalyse the addition of a glycosyl moiety to an acceptor, using an activated sugar as donor (lipid, nucleotide…) [13]. It is considered that GTs are encoded by 1% of total genes, and over 300 000 representatives of GT superfamily have been classified according to their nucleotide sequence into 103 subfamilies [8]. Depending on the conservation of the anomeric atom stereochemistry of the sugar during GT-catalysed reactions, GTs are also classified as inverting or retaining (Figure 1). Inverting GTs operate via a S_N_2 mechanism in a single displacement step where an acid/base residue enhances the nucleophilicity of the acceptor, via an oxocarbenium-like transition state. Unlike inverting GTs, there is much controversy of the molecular mechanism of retaining GTs [13–15]. Retention of the anomeric carbon stereochemistry can occur either following a two-step displacement S_N_2 type mechanism (as in retaining GH), or via a “S_N_i-like” mechanism, that involves a front-side single displacement, both via two distinct oxocarbenium-like transition states [16].

In all three mechanisms, the nucleophilic attack of the acceptor is enhanced by the deprotonation – either by an acid/base residue, like in inverting GT and two-step displacement retaining GT, or the phosphate donor (in an S_N_i-like retaining mechanism). In the case of *O*-GTs, the nucleophile is an alcohol or a phenol (carbohydrates, serine, threonine, …), whereas in *N*-GTs, the nature of the nitrogen-containing group is more diverse (amines, amides, guanidine or even indoles) [12]. *S*- and *C*-GTs follow a similar mechanism with the nucleophilic attack of the acceptor, however, little information is known on the pathways involved in these reactions (S_N_2 or S_N_i-like), because of the few examples of such enzymes characterization in literature, when compared to canonical *O*- and *N*-GTs.

### *S*-Glycosyltransferases

**Figure 2:**
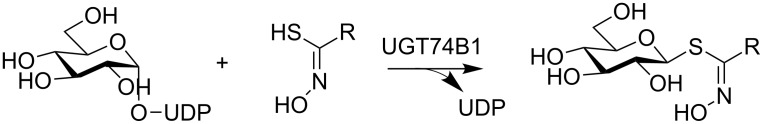
Desulfoglucosinolate biosynthesis by UGT74B1.

Few examples of natural *S*-glycosides have been described in literature [12,17–18]. Historically, glucosinolates have been the first identified *S*-glycosides for 50 years in cruciferous vegetables [19]. Along with the myrosinase GH enzyme, they are part of the “mustard bomb” system as a protective mechanism for plants against insect aggression. Their biosynthetic pathway requires the action of a *S*-GT (UGT74B1) that catalyses the reaction between a thiohydroximate acceptor and UDP-α-D-glucose as sugar donor to yield the corresponding desulfoglucosinolate (Figure 2) [20–21]. UGT74B1 from *A. thaliana* is a versatile enzyme in terms of sugar donor scope, and our group has shown the potency of this enzyme as a biocatalyst for the chemoenzymatic synthesis of non-natural desulfoglycosinolates [22]. More recently, *S*-glycosylated peptides have been identified, and characterized. In bacteria the structures of sublancin [23], glycocin F [24–25], and thurandacin [26] revealed *S*-glycosylation of cysteines. Carbohydrates bound to these bacteriocins are glucose or *N*-acetylglucosamine. For two of these glycopeptides, the corresponding *S*-GTs have been characterized and their versatility for a wide range of sugar donors has been tested [26–27]. More recently, a global protein glycosylation analysis through chemical labelling and mass finger printing have identified many *S*-glycosylation sites on different proteins, with a *N*-acetylglucosamine group bound on cysteines [28].

**Figure 3:**
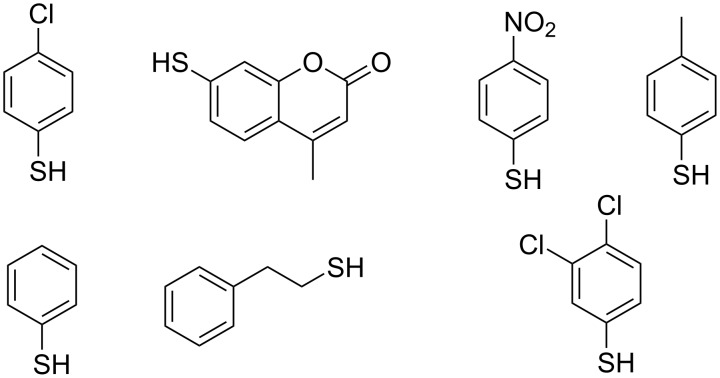
Examples of thiol-containing acceptors used in the chemo-enzymatic biosynthesis of *S*-glycosides catalysed by *S*-GT.

Other enzymes have been scarcely identified to catalyse the *S*-glycosylation, although their endogenous role is not to generate *S*-glycosides. Brazier-Hicks and colleagues have screened many *A. thaliana* Family 1 GTs with three acceptors, to identify *O*-GT, *N*-GT and *S*-GT enzymatic activities [29]. Among the 99 enzymes tested, 17 were able to use 4-chlorothiophenol as the acceptor. UGT74B1, involved in glucosinolate biosynthesis (see supra), was one of these 17 enzymes. Other studies have identified *S*-GT activities when assaying the catalytic promiscuity of *O*-GT with a wide range of aglycone acceptors (Figure 3). OleD from *Streptomyces antibioticus* has been the first reported *O*-GT to catalyse *S*-glycosylation on thiol acceptors [30]. Genetic engineering of this enzyme has also led to *S*-GT activities on several thiols. UGT73AE1 from *Carthamus tinctorius* was able to transfer glucose on a wide range of acceptors, including a *S*-containing compound, dichlorothiophenol [31]. More recently, *Bc*GT1 from *Bacillus subtilis* was shown to efficiently catalyse the glucosylation of thiol-containing acceptors [32].

### *C*-Glycosyltransferases

**Figure 4:**
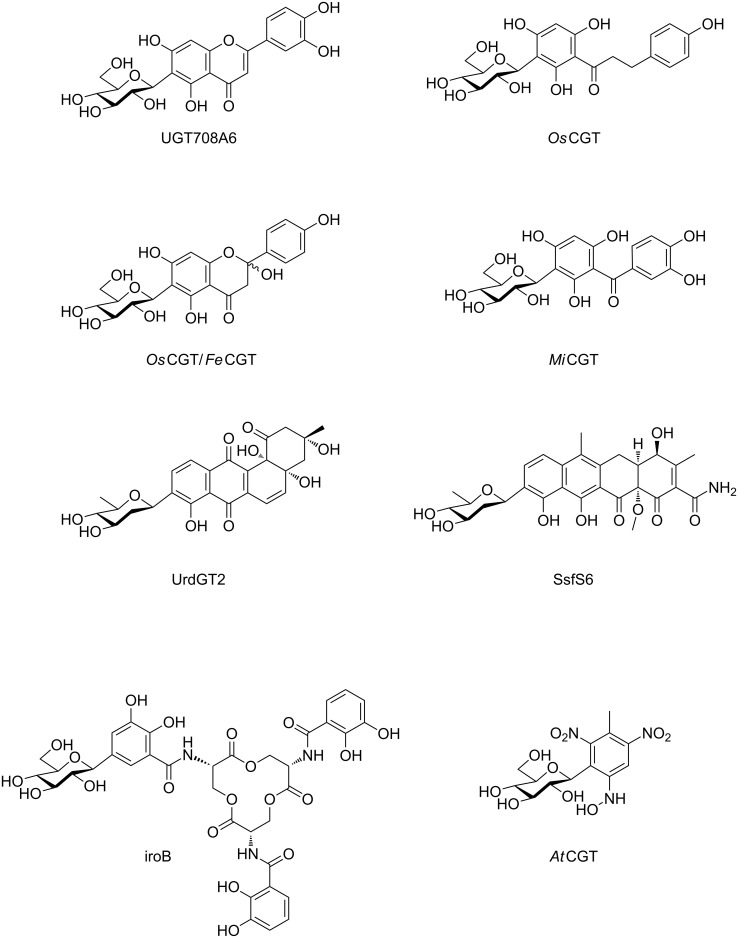
Examples of *C*-glycosylated products biosynthesized by natural *C*-GT. Compounds showed are formed by the action of *C*-GT found in maize (UGT708A6), rice (*Os*CGT), buckwheat (*Fe*CGT), *Mangifera indica* (*Mi*CGT), *Arabidopsis thaliana* (*At*CGT), fungi (UrdGT2 and SsfS6) or bacteria (iroB).

For more than 50 years, *C*-glycosides have been identified in plants [33–34] as secondary metabolites. At least 5 families of aromatic aglycones have been reported to be *C*-glycosylated: flavones, xanthones, chromones, anthrones, and gallic acids. Several corresponding plant *C*-GT have been cloned, expressed and characterized, from several crops including maize [35], rice [36–37], wheat [36], buckwheat [38] and other plants such as *Arabidopsis* [39] or *Mangifera indica* [40] (Figure 4). Fungi *C*-glycosyltransferases were also identified in *Streptomyces*, including UrdGT [41–42] and SsfS6 [43] that catalyse the transfer of the unusual D-olivosyl carbohydrate moiety on the aglycon acceptor. Bacterial *C*-GTs are the last group identified in *Salmonella enterica* and *Escherichia coli* [44–46] that are involved in the biosynthesis of siderophores, that were shown to be *C*-glycosylated enterobactins. In addition to these naturally occurring *C*-GTs, engineering of *O*-GT to *C*-GT were successfully performed in several studies [37,47–48], and chemoenzymatic syntheses of *C*-glycosides were described in other publications [40,49–51]. In all described *C*-GTs, the aglycone acceptor was found to be a derivative of polyhydroxybenzaldehyde, that exhibit an acidic carbon on the aromatic ring. Depending on the nature of the substrate and the *C*-GT involved in the enzymatic reaction, several regioselectivities were observed. A mechanistic study in 2013 by Gutmann and Nidetzky demonstrated that *C*-glycosylation occurred through a direct nucleophilic attack of an acidic carbon, and showed evidence against an *O*-glycosylation followed by an *O*-to-*C* rearrangement [37]. A last family of *C*-GT are the enzymes involved in *C*-mannosylation of protein tryptophanes [37]. However, if the corresponding *C*-GTs were identified, no mechanistic evidence was reported to date [52].

### Glycoside hydrolases

**Figure 5:**
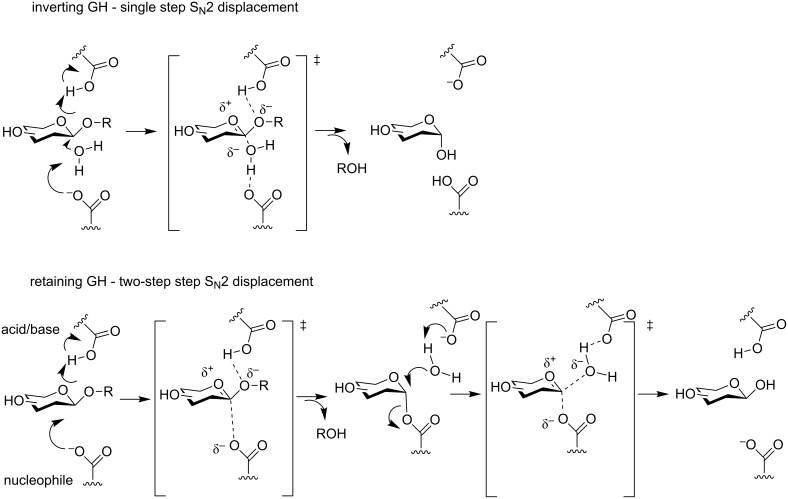
General mechanisms of the *O*-GHs-catalysed hydrolysis.

GHs (E.C. 3.2.1.x) are ubiquitous enzymes responsible for the hydrolysis of the carbohydrate moieties in all the living organisms. They are actually classified in the CAZY database under 145 families, which contain more than 435,000 individual proteins [8]. Like the mechanisms described for the GTs, the catalysis of the hydrolytic reaction can occur with inversion or retention of configuration (Figure 5) as first described by Koshland [53]. However, in the case of GHs, the mechanism generally implies the intervention of two amino acid side chains, typically glutamate or aspartate, and goes through oxocarbenium ion-like transition states. Inverting GHs operate via a single step S_N_2 displacement using a general acid and a general base assistance from two amino acid side chains located 6 to 11 Å apart. The mechanism of retaining GHs occurs via a two-step S_N_2 displacement involving a glycosyl–enzyme intermediate, with the assistance of an acid/base and a nucleophile through two amino acid side chains located 5.5 Å apart.

**Figure 6:**
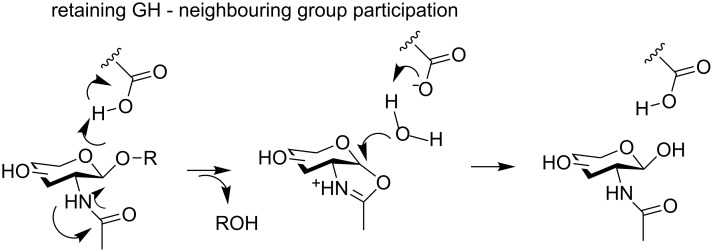
Neighbouring group participation mechanism of retaining the *O*-GHs-catalysed hydrolysis.

A particular case of the retaining GHs is the one of the *N*-acetyl-β-hexosaminidases from the families 18, 20, 25, 56, 84 and 85 in which there is no catalytic nucleophile but where the *2*-acetamido group of the substrate is acting as an internal nucleophile (Figure 6) [54].

Rarer mechanisms have also been discovered, like for example the one of i) myrosinases in plants that are retaining GHs that lack a general acid and use an exogenous base [55], or ii) some GHs from families 4 and 109 which follow a NAD-dependent hydrolysis [56–57].

Even if the major activity of GHs remains the hydrolysis, since the 1970’s, these enzymes have also demonstrated their capacities to catalyse the formation of new glycosidic *O*-linkages, either by reverse hydrolysis or through transglycosylation reactions. First discovered by the team of Bourquelot and Viebel, the reaction of reverse hydrolysis can occur in the presence of GHs when a nucleophile other than water is present in the media [58–59]. Under thermodynamic control, it generally leads to the major formation of the hydrolytic product. More interestingly and in the case of the retaining GHs, the glycosyl–enzyme intermediate can be attacked by another nucleophile than water (like an alcohol) to stereospecifically yield a new glycoside [60]. The reaction is now under kinetic control and the enzyme is named a transglycosidase. The rules that guide the balance between hydrolysis and transglycosylation are still not well understood and controlling this ratio remains a challenge that still need to be solved, even if the use of artificial donors [10], the bioengineering of these biocatalysts [61] and the study of internal water dynamics [62] for examples have permitted important progresses. Consequently, such enzymatic approaches can nowadays efficiently be utilized in particular for the preparation of pure and well-defined complex glycoproteins [63].

### Use of external nucleophiles

The identification of the two amino acid side chains in both retaining and inverting GHs is usually performed through site directed mutagenesis of the potent residues [64]. In these cases, the mutated enzymes are no longer able to perform the hydrolysis of the substrates. The use of external and suitable nucleophilic anions such as azide, formate or acetate allows the rescue of the activity and can also represent an efficient methodology of enzymatic synthesis of these particular (but mostly unstable) carbohydrate derivatives. These mutants were also developed as powerful biocatalysts for the synthesis of complex *O*-glycosides through the concepts of glycosynthases or thioglycosynthases [61].

### *S*-Glycoside synthesis

**Figure 7:**
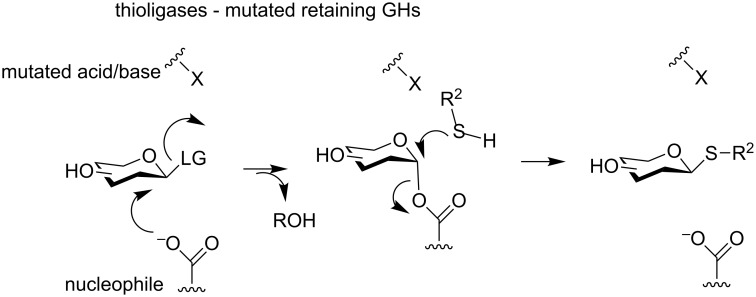
Mechanism of the thioligases.

In retaining GHs, the inactivation of the acid/base catalytic residue is of particular interest, and can lead to an original biocatalyst with poor hydrolytic activities but the ability to promote the formation of thioglycosidic linkages (Figure 7). Such mutated enzymes were firstly described by the team of Withers and are named thioligases [65]. Based on the mechanism, here the formation of the glycosyl–enzyme intermediate requires the use of an activated glycosyl donor, such as dinitrophenyl or azide glycosides, and the glycosylation step needs stronger nucleophiles such as thiol derivatives. The choice of the amino acid to mutate the acid/base is of crucial importance as it directly dictates the level of activity [66], but it cannot be predicted nor be a guarantee for success [67].

**Figure 8:**
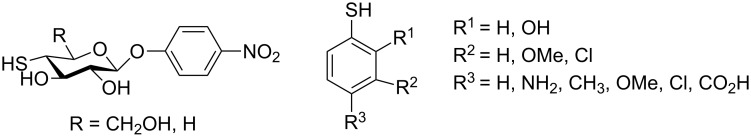
Examples of thiol acceptors utilized with GHs.

In general, the reported thiol acceptor is a monosaccharide or a substituted thiophenol (Figure 8). These engineered GHs were already successfully applied to the biocatalysed synthesis of thiodi- or -trisaccharides [68–72], neo-thioglycoprotein [73], or even simple thioglycosides with potent inhibitory properties [74]. More complex biomolecules were also obtained like glycans or glycopolymers [75], or even rarer thiofuranosides [76].

### *C*-Glycosides synthesis

There is no example of *C*-glycoside synthesis promoted by GHs reported in the literature so far, although as depicted by the mechanism, this kind of biocatalysed reaction can be envisioned.

## Conclusion

To conclude, the enzymatic mechanisms that rule the activities of GTs and GHs begin to be well understood by the glycoscientists. Their application to the enzymatic synthesis of a great variety of *O*- and *N*-glycosides are already becoming a routine. In addition the utilization of enzymes so to obtain rarer *C*- and *S*-analogues is an emerging field restricted to few acceptors (Table 1). Still, as demonstrated by the CAZY database, the glycoscientists have nowadays access to a large (and increasing) library of GTs and GHs, and in a near future, they will be able to perform most reactions enzymatically. In addition, the access to large quantities of inexpensive substrates can also be envisioned. In parallel, our knowledge of the enzymatic mechanisms has allowed us to modify and improve the original activities through reasoned site-directed mutagenesis. However, despite major advances, all the rules that finely tune the biocatalysts are still poorly understood and the luck is in too many cases the best road to success. The unlock of the biotechnological bolts in this particular field will certainly occur by our deep understanding of the role of second-sphere amino acids and of protein motions. This will require the generation of huge libraries of mutants and the fast screening of their activities, as well as powerful molecular modelling and crystallization of proteins. No doubt then that such biocatalysts will represent a competitive tool for glycosylation so to obtain complex *O*-, *S*- or *C*-glycoconjugates of biological interests.

**Table 1:** Summary of rare synthetic activities of carbohydrate-related enzymes.

Activity	Enzyme family	Acceptor	References

*S*-glycosylation	GTs	desulfoglycosinolates	[20–22]
		cysteine	[26–28]
		aromatics	[29–32]
	GHs	saccharides	[68–73]
		aromatics	[74–76]
*C*-glycosylation	GTs	aromatics	[35–51]
	GHs	no reference	no reference
